# LncRNA and mRNA Expression Profiles in Methylprednisolone Stimulated Neural Stem Cells

**DOI:** 10.3389/fnins.2021.669224

**Published:** 2021-06-23

**Authors:** Yong Tang, Zhongyu Xie, Mengjun Ma, Kaidi Duan, Yuxi Li, Jichao Ye

**Affiliations:** ^1^Department of Orthopedics, Sun Yat-sen Memorial Hospital, Sun Yat-sen University, Guangzhou, China; ^2^Department of Orthopedics, The Eighth Affiliated Hospital, Sun Yat-sen University, Shenzhen, China

**Keywords:** neural stem cells, methylprednisolone, long non-coding RNA, mRNA, spine injury

## Abstract

Spinal cord injury (SCI) is a devastating neurological disorder that affects thousands of individuals each year. Previously, our study in non-human primates with SCI demonstrated that methylprednisolone (MP) resulted in the dysfunction of neural stem cells (NSCs), which may help to explain the controversial roles of MP in SCI. However, the detailed mechanism is still unclear. In this manuscript, we investigated the LncRNA and mRNA expression profiles of NSCs treated with MP. A total of 63 differentially expressed LncRNAs and 174 differentially expressed mRNAs were identified. Gene ontology (GO) analysis showed that differentially expressed mRNAs were highly associated with terms related to regulation of external stimulation, secretion, and migration. Kyoto Encyclopedia of Genes and Genomes (KEGG) analysis results indicated that the PI3K–Akt signaling pathway contributed to the functions of MP treated NSCs. Besides, 3899 co-expression pairs were constructed among the differentially expressed LncRNA and mRNA, among which five predicted target mRNAs with the differentially expressed LncRNAs were identified. These results provide greater insight into the precise mechanisms of MP mediating NSC dysfunction in SCI.

## Introduction

Spinal cord injury (SCI) is a devastating neurological disorder that affects thousands of individuals each year. As about 150–180 hundred new cases increasing every year, the amount of the SCI patient population was estimated to be over 3 million cases around the world (David et al., 2019). However, not only the clinical treatment but also the functional rehabilitation is still the highly problematic issue in clinic, which greatly affects the physiological, psychological, and social activity of the SCI patients (Silva et al., 2014).

Methylprednisolone (MP), a kind of glucocorticoid, is used to be one of the most important medicine in SCI treatment at acute phase. Although several randomized controlled trial studies demonstrated the effectiveness of MP in SCI treatment (Bracken et al., 1984, 1990, 1997), more and more controversies arose recently because more complications but less effectiveness of MP were observed in patients with SCI (Evaniew et al., 2016). Previously, our study in non-human primates with SCI demonstrated that MP resulted in the dysfunction of neural stem cells (NSCs), which may help to explain the controversy of MP in SCI (Ye et al., 2018). NSCs are a kind of adult pluripotent stem cells located in nervous tissue *in vivo* (Ortiz-Alvarez et al., 2019). NSCs can differentiate into multiple cell lineages including neuron, astrocyte, and oligodendrocyte, which greatly contribute to nervous tissue repairment and regeneration (Tang et al., 2017). However, the molecular mechanism that MP affects the functions of NSCs is still unclear.

Long non-coding RNA (LncRNA), a novel member of non-coding RNA, is characterized by the length larger than 200 nt and without protein-coding potential (Ransohoff et al., 2018). Researches have demonstrated that LncRNA played an important role in regulation of cell function, which was greatly involved in physiological and pathological processes *in vivo* (Gil and Ulitsky, 2020). Moreover, it has been reported that LncRNA expression was up- or downregulated after glucocorticoid stimulation (Bunch, 2018). Whether LncRNAs contribute in the MP mediating NSC dysfunction needs to be addressed.

In this study, we investigate the expression profiles of LncRNA and mRNA of NSCs treated with MP using a microarray, followed by bioinformatics analysis. These results provide greater insight into the precise mechanisms of MP mediating NSC dysfunction in SCI.

## Materials and Methods

### Ethics Statement

This study was approved by the Committee for the Care and Use of Laboratory Animals of Sun Yat-sen University, Guangzhou, China. This study was performed in the Laboratory Animals Center of Sun Yat-sen University.

### Isolating and Culturing NSCs

To isolate the NSCs from mice, neonatal C57/BL male mice were sacrificed through overdose of anesthesia. The spinal cord tissues of the neonatal C57/BL mice were separated and then thoroughly cut into pieces in a sterile environment. A total of 3 ml DMEM/F12 medium supplemented with 2% B27 supplement, 5 μg/ml heparin, 20 ng/ml bFGF, and 20 ng/ml EGF were added to resuspend the tissue. These suspensions were seeded in a 25-cm^2^ culture flask and cultured at 37°C humidified atmosphere with 5% CO_2_. Medium was changed every 3 days, and the adherent cells were removed. Suspended neural spheres containing NSCs were used for experiments.

### MP Stimulation

Suspended neural spheres were centrifuged at 1,500 rpm, and the supernatant was removed. Trypsin (0.25%) supplemented with 0.53 mM EDTA was used to digest the neural spheres into single cell NSCs, which were then seeded in a six-well plate at a density of 2 × 10^4^ cells/cm^2^ using culture medium. MPs were added in the culture medium at a concentration of 10 μg/ml according to our previous study (Wang et al., 2014). Culture medium without MP was used as a control. After 24 h stimulation, NSCs were collected for experiments described below.

### RNA Isolation and Reverse Transcription

Three MP treated NSCs and control NSCs were collected as described above. Total RNA of NSCs was extracted using Trizol reagent and was then purified using NucleoSpin^®^ RNA clean-up kits according to the instructions. RNA integrity was determined by formaldehyde denaturing gel electrophoresis. The purity and concentration of RNA were determined from OD260/280 readings using spectrophotometer. Total RNA was reverse transcribed into cDNA, which was successively labeled with fluorescent dye (Cy3-dCTP) and hybridized with LncRNA + mRNA Gene Expression Microarray V4.0 (4 × 180 K; CapitalBio Corp).

### Microarray Detection and Analysis

The microarrays prepared above were washed and scanned using G2565CA Microarray Scanner. Data were analyzed for data summarization, normalization, and quality control by using the GeneSpring software (version 13.0). Differentially expressed genes were identified according to the criterion of fold change > | 2.0| and *P-*values < 0.05.

### Bioinformatics Analysis

Functional enrichment of the differentially expressed genes was performed using the Gene ontology (GO) analysis. The signaling pathways with statistic difference were identified using the Kyoto Encyclopedia of Genes and Genomes (KEGG) database based on the differentially expressed genes. Coding–non-coding gene co-expression (CNC) networks were constructed based on the results of correlation analysis between mRNA and LncRNA expression, which meets the criterion of Pearson correlation coefficients > | 0.99| and *P*-values < 0.05. Based on the CNC network, the sequences of LncRNA and mRNA were compared and analyzed to identify the predicted target differentially expressed mRNA of the differentially expressed LncRNA.

### Quantitative Real-Time Polymerase Chain Reaction (qRT-PCR)

RNAs of nine NSCs were isolated and reverse transcribed into cDNA. QRT-PCR was performed using SYBR Premix Ex Taq in the LightCycler 480 PCR System. All the data were normalized by GAPDH. The relative expression levels of LncRNA and mRNA were analyzed using the 2^–△^^△^^*Ct*^ formula. The forward and reverse primers for each gene were present in Supplementary Table 1.

### Statistical Analysis

Statistical analysis was performed with SPSS software (version 24). Data were expressed as means ± SD, and *P*-values < 0.05 were considered as statistically significant difference.

## Results

### MP Altered the LncRNA and mRNA Expression Profile of NSCs

**FIGURE 1:**
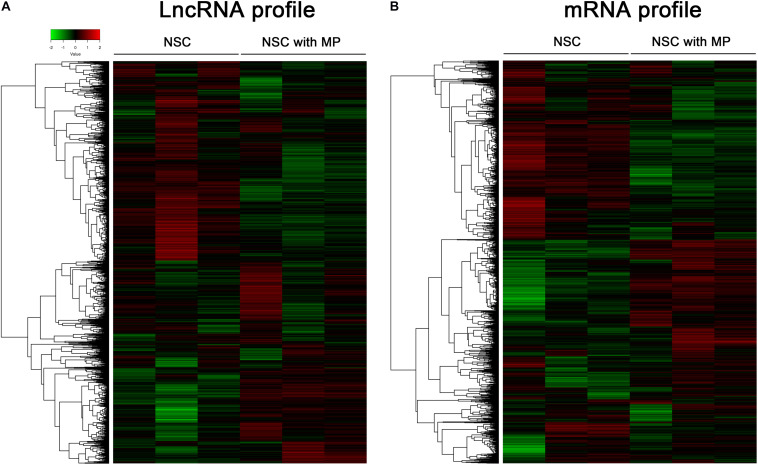
Clusters of differentially expressed LncRNA and mRNA of NSCs. **(A)** A total of 63 LncRNAs were differentially expressed in MP treated NSCs (*n* = 3) compared with control NSCs (*n* = 3). Hierarchical clustering showed a distinguishable LncRNA expression profile. **(B)** A total of 174 mRNAs were differentially expressed in MP treated NSCs (*n* = 3) compared with control NSCs (*n* = 3). Hierarchical clustering showed a distinguishable mRNA expression profile.

Mouse NSCs with MP stimulation at a concentration of 10 μg/ml for 24 h as well as control NSCs were detected using a LncRNA–mRNA microarray. Results showed that a total of 63 differentially expressed LncRNAs were identified, which contained 39 upregulated LncRNAs and 24 downregulated LncRNAs in MP stimulated NSCs compared to control NSCs (Figure 1A). The top 10 LncRNAs with the largest significant difference are shown in Table 1. Moreover, there were 174 differentially expressed mRNAs in MP stimulated NSCs compared to the control group, including 89 upregulated mRNAs and 85 downregulated mRNAs (Figure 1B). The top 10 mRNAs with the largest significant difference are shown in Table 2. These results indicated that MP treatment altered the LncRNA and mRNA expression profiles of NSCs.

**TABLE 1:** The top 10 differentially expressed LncRNAs.

LncRNA ID	Fold change	Regulation	Chromosome	Strand	Start	End	Length (bp)
NONMMUT028948	4.773249	Up	17	–	28557020	28559662	2644
NONMMUT037684	4.21413	Up	2	+	2963595	2968499	4920
NONMMUT040417	3.917528	Up	4	–	3610574	3615192	3395
NONMMUT067035	3.822269	Down	8	+	2119838	2124712	4874
NONMMUT028949	3.691979	Up	17	–	28559406	28561336	1932
NONMMUT018528	3.428622	Down	13	–	84484213	84488784	4445
NONMMUT039150	3.387517	Down	2	–	3856163	3859020	2761
NONMMUT051172	3.214711	Up	19	–	686750	689491	2766
NONMMUT037015	3.196916	Down	11	+	4472683	4474874	2196
NONMMUT047659	3.188095	Up	4	+	65017170	65019384	2215

**TABLE 2:** The top 10 differentially expressed mRNAs.

Gene symbol	Fold change	Regulation	Chromosome location
*Lce1e*	8.522058	Down	chr3:92511424–92511365
*Lce1i*	7.390228	Down	chr3:92581218–92581159
*Itga2*	7.223627	Down	chr13:115626773–115626714
*Sulf1*	7.149574	Down	chr1:12849507–12849566
*Sult1a1*	6.932767	Up	chr7:133816716–133816657
*Orm2*	6.452506	Up	chr4:63024979–63025038
*Bcl2a1d*	6.34652	Down	chr9:88618224–88618165
*Galnt15*	5.318896	Up	chr14:32871453–32871512
*Slamf6*	5.315329	Down	chr1:173871767–173871826
*Sult1a1*	5.229404	Up	chr7:133816664–133816605

### GO Analysis of Differentially Expressed mRNA of MP Stimulated NSCs

**FIGURE 2:**
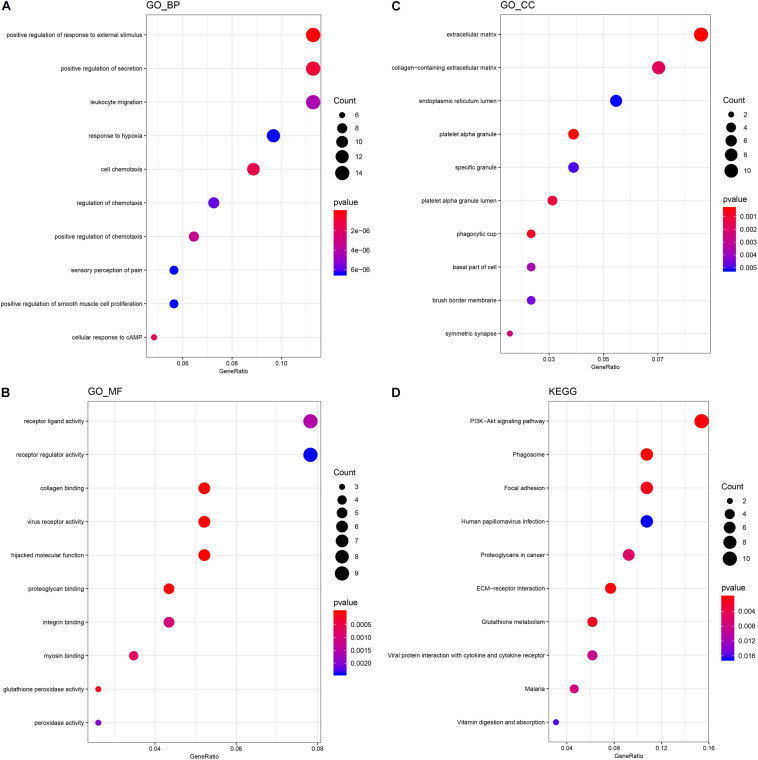
GO and KEGG analysis of differentially expressed mRNA. **(A)** The top 10 GO terms of the biological process domain. **(B)** The top 10 GO terms of the molecular function domain. **(C)** The top 10 GO terms of the cellular component domain. **(D)** The top 10 signaling pathways enriched by the KEGG database.

In order to speculate the possible function of NSCs affected by MP stimulation, GO analysis was performed. All differentially expressed mRNAs were classified into three domains, including biological process, molecular function, and cellular component. In the biological process domain, four GO terms of the top 10 GO terms for the differentially expressed mRNAs were about cell migration. Besides, response to external stimulus and secretion regulation were enriched in the top 10 GO terms of biological process domain (Figure 2A). In the molecular function domain, the top 5 GO terms were receptor ligand activity, receptor regulator activity, collagen binding, virus receptor activity, and hijacked molecular function (Figure 2B). Moreover, the top 5 GO terms of cellular component domain were extracellular matrix, collagen-containing extracellular matrix, endoplasmic reticulum lumen, platelet alpha granule, and specific granule (Figure 2C).

### KEGG Analysis of Differentially Expressed mRNA of MP Stimulated NSCs

KEGG analysis was then performed to further speculate the functional signaling pathways of NSCs affected by MP stimulation. A total of 22 signaling pathways were enriched by KEGG analysis based on the differentially expressed mRNA. The top 5 signaling pathways in KEGG analysis are shown in Table 3. Among these signaling pathways, the PI3K–Akt signaling pathway was the largest significant difference pathway with the highest gene ratio (differentially expressed gene/total gene in pathway) (Figure 2D), indicating that the PI3K–Akt signaling pathway has an important role in MP stimulated NSCs.

**TABLE 3:** Top 5 pathways with largest significant difference in KEGG analysis.

Pathway	Count	*P* value	Gene
Phagosome	7	0.000213	*THBS1*, *MRC1, THBS2*, *CTSL*, *CYBB*, *CORO1A*, *ITGA2*
PI3K–Akt signaling pathway	10	0.000507	*THBS1*, *THBS2*, *PDGFD*, *FGF18*, *DDIT4*, *IBSP*, *IGF1*, *TNC*, *GNGT2*, *ITGA2*
ECM–receptor interaction	5	0.0007	*THBS1*, *THBS2*, *IBSP*, *TNC*, *ITGA2*
Glutathione metabolism	4	0.001054	*MGST2*, *GPX3*, *GSTM2*, *ANPEP*
Focal adhesion	7	0.001076	*THBS1*, *THBS2*, *PDGFD*, *IBSP*, *IGF1*, *TNC*, *ITGA2*

### Predicted Target mRNA and Related Differentially Expressed LncRNA of MP Stimulated NSCs

**FIGURE 3:**
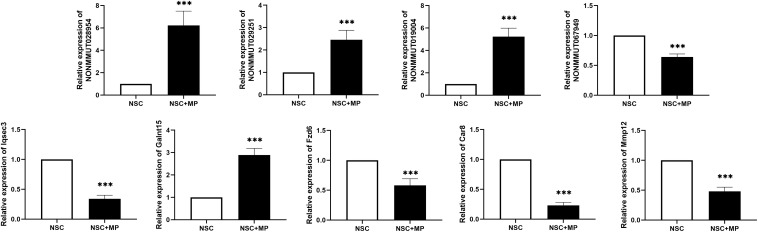
Differentially expressed LncRNA and mRNA confirmation by qRT-PCR. Five pairs of the differentially expressed LncRNAs and their predicted target mRNAs were confirmed by qRT-PCR. The mRNAs *Iqsec3*, *Fzd6*, *Car8*, and *Mmp12* were downregulated, and *Galnt15* was upregulated. NONMMUT067949 was downregulated, and NONMMUT029251, NONMMUT019004, and NONMMUT028954 were upregulated. *N* = 9, ***P < 0.001.

A CNC network was constructed to clarify the relationship of the differentially expressed LncRNA and mRNA. There were 3899 co-expression pairs identified among the differentially expressed LncRNA and mRNA. Based on this CNC network, we investigated and identified five pairs of the differentially expressed LncRNA and their predicted target mRNA (Table 4). NONMMUT029251 and NONMMUT067949 positively regulated their target mRNA *Galnt15 and Mmp12* separately, and NONMMUT028954 and NONMMUT028954 negatively regulated their target gene. The predicted regulatory mechanisms were *cis*-acting (four pairs) or as trans-acting (one pair). Moreover, the expression level of these LncRNA and mRNA was confirmed by qRT-PCR, which is consistent with the results of microarray (Figure 3).

**TABLE 4:** Differentially expressed LncRNA and predicted target gene.

LncRNA ID	Gene symbol	Correlation value	*P* value	LncRNA regulation	mRNA regulation	Predict mechanism
NONMMUT067949	*Mmp12*	0.981205	0.000527	Down	Down	*Cis*-acting
NONMMUT029251	*Galnt15*	0.991102	0.001118	Up	Up	*Trans*-acting
NONMMUT019004	*Car8*	–0.94825	0.003948	Up	Down	*Cis*-acting
NONMMUT019004	*Fzd6*	–0.9302	0.007137	Up	Down	*Cis*-acting
NONMMUT028954	*Iqsec3*	–0.91941	0.009479	Up	Down	*Cis*-acting

## Discussion

NSCs, a kind of adult multipotential stem cell, are one of the critical cells in nervous tissue repairment after SCI. Once the spine cord injury occurs, endogenous NSCs are firstly activated from quiescent status, followed by migration to the injured sites, and then exhibit their healing function through differentiation into neuron or secreting cytokines (Stenudd et al., 2015). Except for the endogenous NSCs, more and more studies have demonstrated that exogenous NSC transplantation improved the recovery of nervous tissue in SCI models, emphasizing the important role of NSCs in SCI treatment (Yousefifard et al., 2016). However, these reparative processes are widely and usually affected by kinds of factors *in vivo* and *in vitro*.

MP is a widely used glucocorticoid for many diseases in clinic, in which its effectiveness is nevertheless a controversial focus of SCI treatment (Ahuja et al., 2017). Recently, our research found that MP inhibited the proliferation of NSCs in a non-human primate with SCI. Besides, MP affected the differentiation potentials of NSCs in a rat model with SCI (Wang et al., 2014). Moreover, high dose MP after SCI significantly reduces the proliferation rate of NSCs *in vitro* (Schroter et al., 2009). These results remind us that the ineffectiveness of MP in SCI may partially come from its effect on NSCs, but the detailed mechanism is largely unknown.

NSC functions were under the control of kinds of intracellular regulatory mechanisms at gene transcription and protein translation levels, among which LncRNAs were one of the most important mechanisms (Kim, 2016; Ramos et al., 2016). LncRNAs widely exist in cells and regulate the cells’ function through *trans*/*cis*-acting or endogenous competitive RNA (ceRNA) mechanisms (Guil and Esteller, 2015). Previously, many studies have shown the large involvement of LncRNA in regulating functions of NSCs. A LncRNA named Pnky was determined to be a neural-specific LncRNA to inhibit the neuronal differentiation of NSCs (Ramos et al., 2015). Moreover, another LncRNA, LncRNA-158, was proven to promote the NSC differentiation into oligodendrocytes through the NFκB signaling pathway (Li et al., 2018). Nevertheless, the LncRNA expression profiles are easily affected by external stimulation, which in turn results in function alternation of NSCs (Lu et al., 2018). In this study, in order to figure out the role of LncRNA in MP mediating NSC dysfunction, a LncRNA–mRNA microarray was performed to scan their expression in NSCs after MP stimulation. There were 63 differentially expressed LncRNAs and 174 differentially expressed mRNAs identified in MP treated NSCs compared to those control NSCs without stimulation. These results indicated that not only the mRNA expression profile but also the LncRNA expression profile was altered by MP treatment. Moreover, the possibility that MP affected the NSC function through regulation of their LncRNA expression existed.

A cell’s mRNA expression profile reflects its status and function. To investigate the MP affected functions of NSCs, GO was performed to enrich the differentially expressed mRNA. In the biological process domain of GO terms, functions about cell migration, secretion, and stimulation reaction were enriched, suggesting that MP stimulation at a concentration of 10 μg/ml for 24 h mainly affects these functions of NSCs. It is worth noting that NSCs migrating to the sites of injury and secreting large amount of neurotrophic factors are two key steps in neural restoration except for differentiation directly (Stenudd et al., 2015). Therefore, we suggest that MP may also affect the NSC migration and secretion potential in addition to the previously reported ability in proliferation and differentiation. To explore the enriched signaling pathway, we then performed the KEGG analysis based on the differentially expressed mRNA profile. Although 22 signaling pathways were enriched, the PI3K–Akt signaling pathway was determined to be the largest significant difference pathway with the highest gene ratio. It has been widely proven that the PI3K–Akt signaling pathway contributed greatly to the functional regulation of NSCs (Lee et al., 2016; Zhang et al., 2017; Wang et al., 2018). To our knowledge, for the first time, we demonstrated that MP may affect the NSCs by modulating the PI3K–Akt signaling pathway. Song et al. (2017) found that LncRNA named IGF2AS could inactivate the Akt signaling pathway and then protected the NSC derived neurons from apoptosis. We speculated that differentially expressed LncRNA may activate or inactivate the PI3K–Akt signaling pathway, which ultimately caused the dysfunction of NSCs. Functional experiments *in vivo* and *in vitro* should be performed to confirm this hypothesis.

LncRNAs always show a positive or negative correlation in expression level to their target gene (Chen, 2016). To further clarify the relationship and regulatory mechanism of differentially expressed mRNA and LncRNA, we identified the predicted target mRNA and their related LncRNA using bioinformatics methods based on the CNC network of those differentially expressed mRNA and LncRNA in this study. Among the five identified pair, NONMMUT067949-MMP12 was the most remarkable combination. Previous study by Shan et al. demonstrated that MMP12 was a critical molecular in the activation and development of NSCs (Shan et al., 2018). As our results, NONMMUT067949 may be positively related to MMP12 expression. After MP stimulation, NONMMUT067949 expression decreased, which downregulated the expression of MMP12 through *cis*-acting mechanism, and finally resulted in the dysfunction of NSCs. These relationships and the regulatory mechanisms are also need to be addressed in the future study.

In summary, we investigated the LncRNA and mRNA expression profiles of NSCs treated with MP. This study may help to understand the mechanism of MP to regulate NSCs’ function and the role of LncRNA in this process. However, limitations still exist in this study. The detailed regulatory mechanisms of these identified LncRNA are still unknown in cells and mice models. Besides, the LncRNA expression profiles of NSCs treated by MP at different concentrations or stimulation times have not been clarified. Further studies should be addressed to elucidate these questions.

## Data Availability Statement

The data of microarrays can be obtained from the NCBI SRA database at this link: https://www.ncbi.nlm.nih.gov/Traces/study/?acc=PRJNA727403&o=acc_s%3Aa.

## Author Contributions

JY, YT, and ZX designed research studies, conducted the experiments, analyzed the data, and wrote the manuscript. MM and YL conducted the experiments, analyzed data, and wrote the manuscript. KD conducted the experiments. All authors contributed to the article and approved the submitted version.

## Conflict of Interest

The authors declare that the research was conducted in the absence of any commercial or financial relationships that could be construed as a potential conflict of interest.
