# Supplementary material for: LncRNA and mRNA Expression Profiles in Methylprednisolone Stimulated Neural Stem Cells
Source: Front Neurosci. 2021 Jun 23;15:669224. doi: 10.3389/fnins.2021.669224 (PMC8262496; doi:10.3389/fnins.2021.669224)
Supplement: Supplementary file 1 [file Table_1.DOCX]

**Supplemental Table 1 Primers used for PCR**

| **Name** | **Forward primer**  **(5`-3`)** | **Reverse primer**  **(5`-3`)** |
| --- | --- | --- |
| GAPDH | TGACCTCAACTACATGGTCTACA | CTTCCCATTCTCGGCCTTG |
| NONMMUT067949 | AACATCAGGGACATTGACGTG | GCCGTGGCACTTACATTCC |
| NONMMUT029251 | CCTCATCGAACACCCAGACC | CCCTCCTGCTTGGACACAAAG |
| NONMMUT019004 | AGGAGGAGACGTGCGAGAAA | GTGGGTTTCCACCATTAGCAC |
| NONMMUT028954 | CAGCACACTCGATATGGACCA | GCGAGTAATAGCGTGGACTAC |
| Mmp12 | GGGCTGCTCCCATGAATGAC | CCAGAGTTGAGTTGTCCAGTTG |
| Galnt15 | GGAGCCTCAAGACCATGTGC | GGTGTCCTCTTGTTCGGCTT |
| Car8 | ATGGCTGACCTGAGCTTCATT | ACCTTCCTCGTAACCCCACT |
| Fzd6 | TCTGCCCCTCGTAAGAGGAC | GGGAAGAACGTCATGTTGTAAGT |
| Iqsec3 | GCTGCCTCCGAATACGAACTC | CGAAAAGCGGTTTGAATGGTG |
